# Case Report: Multilevel spondylectomy for en bloc resection of an upper cervical chordoma: surgical technique and conceptualization through the CLiMR framework

**DOI:** 10.3389/fonc.2026.1877083

**Published:** 2026-07-24

**Authors:** Alexander T. Yahanda, Karan Joseph, Samuel Vogl, Ajay Chatrath, Matthew L. Goodwin, Patrik Pipkorn, Camilo A. Molina

**Affiliations:** 1 Department of Neurological Surgery, Washington University School of Medicine in St. Louis, St. Louis, MO, United States; 2 Department of Orthopedic Surgery, Washington University School of Medicine in St. Louis, St. Louis, MO, United States; 3 Department of Otolaryngology, Washington University School of Medicine in St. Louis, St. Louis, MO, United States

**Keywords:** 3D printed implants, cervical chordoma, CLiMR framework, en bloc resection, spinal oncology, spinal tumor classification, spondylectomy

## Abstract

**Introduction:**

Spinal chordomas are locally aggressive primary malignancies of bone that require en bloc surgical resection to optimize outcomes. A rare subset of spinal chordomas occur in the cervical spine, some of which involve the upper cervical spine and craniovertebral junction (CVJ). These tumors require highly complicated and risk-prone surgeries. We report the case of a high-cervical chordoma which was resected en bloc via a three-stage surgical procedure and characterize the case through the novel CLiMR (Column, Line-of-Sight, Margins, Ring) framework for en bloc spondylectomies.

**Case description:**

A 66-year-old male presented with right-sided neck and facial pain. He was found to have a chordoma originating from C2 involving both C3 and the craniovertebral junction. He underwent a posterior-anterior-posterior three-stage surgical resection under the same anesthesia, which lasted approximately 29 hours. Postoperatively, he required prolonged ventilator weaning but is recurrence free and full strength at over two years of follow-up. The CLiMR score for this case was 15.5, which is an exceptionally high score and underscores the anatomic and procedural complexities inherent to the CVJ.

**Conclusion:**

We present the successful en bloc resection of an upper-cervical chordoma as performed through a three-stage operative case. The CLiMR system can provide a standardized tool for operative characterization and risk communication, which can aid in the planning and execution of complex spinal oncology cases.

## Introduction

Chordomas are rare primary tumors of bone (incidence of ~1 in 1 million) that arise from either the skull base or spine (1). Comprising 4% of all malignant bone tumors, they develop from embryonic remnants of the notochord or from benign notochord tumors that undergo malignant transformation. In the spine, chordomas are most commonly found in the sacrum, which account for 50-60% of all chordoma cases. Only ~10% of chordomas are located in the cervical spine (1).

Chordomas are slower-growing but locally aggressive tumors that may metastasize most commonly to the lungs, liver, or other bones. For spinal chordomas, as with other primary osseous spinal malignancies, en bloc surgical resection with negative margins (followed by proton beam or carbon ion radiotherapy) is considered gold standard treatment to minimize the risk of local recurrence and maximize overall survival (2–4). These en bloc resections are technically demanding and highly complex procedures. This is particularly true in the upper cervical spine, which contains critical neurovascular structures and limited reconstruction strategies (5–8). Indeed, prior reports of high cervical chordoma resections have demonstrated higher complication rates and worse incidence of negative margins (4, 9).

The authors of this study previously developed a novel classification system for en bloc spondylectomies that may assist in tumor classification and operative planning (10). This framework – termed the Column, Line-of-Sight, Margins, Ring (CLiMR) framework – incorporates technical complexity and morbidity to promote standardized assessment of primary spinal tumors, augment surgical decision-making and communication, and provide an overview of surgical risk and postoperative outcomes. Herein, we present the case of an upper cervical chordoma involving the craniovertebral junction (CVJ) which was resected en bloc through a three-stage procedure and reconstructed using a custom three-dimensional (3D) printed implant (3DPI). We furthermore apply the CLiMR framework to this tumor to more thoroughly characterize its anatomic and surgical intricacies.

## Case presentation

At time of presentation, the patient was a 66-year-old male with a past medical history of polymyalgia rheumatica, giant cell arteritis, sleep apnea, depression, and hypertension who had been experiencing worsening neck pain that had been slowly progressing over the preceding few years. The pain would intermittently radiate to the right side of his face. He otherwise denied any neurological deficits, including numbness, weakness, cranial nerve dysfunction, dysphagia, difficulties with ambulation, or signs/symptoms of myelopathy. He was neurologically intact on exam.

**Figure 1:**
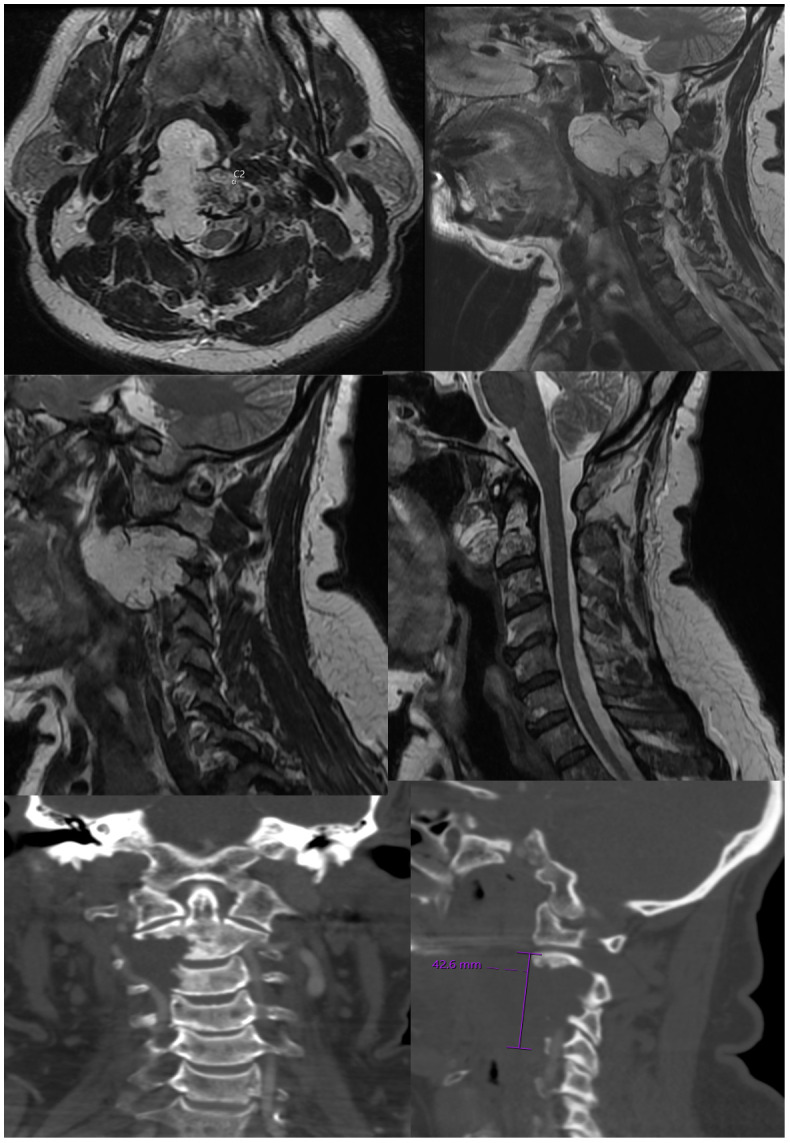
Preoperative imaging, including T2-weighted axial (upper left) and sagittal (upper right, middle images) MRI demonstrating a T2 hyperintense lesion originating from C2. CT angiogram shows encasement and displacement of the right vertebral artery (bottom left) and osseous destruction of C2 and C3 (bottom right).

Cervical spine MRI demonstrated an exophytic T2 hyperintense, heterogeneously enhancing mass centered in the C2 vertebral body measuring 4.5x4.6 cm (**Figure 1**). This mass extended along the right posterior elements with destruction of the right transverse process and right C2 facets. It furthermore extended through the right neuroforamen at C2–3 with epidural extension causing right-sided spinal canal stenosis. Inferiorly, the mass involved the lateral C3 vertebral body, abutting the right C3 transverse process and extending into the right C3–4 neuroforamen. Prevertebral extension measured approximately 2.7 cm with mass effect on the right posterolateral aspect of the oropharynx/nasopharynx. The distal right V2 and proximal V3 vertebral artery segments were encased by tumor with resultant narrowing of the V3 segment, though CT angiogram (CTA) demonstrated patency of the vertebral artery (**Figure 1**). Subsequent PET scan demonstrated moderate tumor FDG avidity and no evidence of metastatic disease. The patient underwent a sheathed needle biopsy of the lesion, which yielded a diagnosis of chordoma.

The patient presented to Neurosurgery clinic to discuss management of his tumor. Given the diagnosis, the decision was made to proceed with en bloc resection to minimize the chances of recurrence. Preoperatively, he underwent catheter angiography to assess blood flow through the vertebral arteries, intracranial vasculature collaterals, and vascular supply to the cervical lesion. Balloon test occlusion demonstrated co-dominance of the vertebral arteries with limited anterior circulation collaterals to the posterior circulation. The patient tolerated balloon test occlusion of the vertebral arteries well.

### Surgical management

The overall surgical plan required three stages (two posterior approaches, one anterior approach), all performed under the same anesthesia. Stage 1 involved a posterior approach for initial tumor mobilization. The patient was positioned prone in the standard fashion. Laminectomies were performed from C1-C4, followed by left facetectomies at C2–3 and C3-4, a left C2 pediculectomy, and a right C3–4 facetectomy. The bilateral C1–2 facets were mobilized with division of the C1–2 capsular ligament. The bilateral C2 nerve roots and the right C4 nerve root were ligated proximal to the dorsal root ganglion and divided. The left vertebral artery was skeletonized from the C2 transverse foramen to the C3 transverse foramen. The right vertebral artery was skeletonized, ligated at both the sulcus arteriosus and the level of the C4 transverse foramen, and divided. At completion, the patient was closed and turned supine for stage 2.

Stage 2 involved an anterior cervical approach for mobilization of the anterior component of the tumor and en bloc tumor delivery. This was performed in conjunction with an otolaryngology co-surgeon. A high cervical Smith-Robinson approach and submucosal neck dissection were performed. A right mandibulectomy was required to expose the anterior cervical spine from the clivus to C5. A C3–4 discectomy and bilateral uncinectomies were performed, and the bilateral vertebral arteries were further skeletonized anteriorly. The right C3 nerve root was ligated proximal to the dorsal root ganglion and cut. Rostral osteotomies were performed in C3 just medial to the uncinate processes, extending up to the C2–3 disc space without breaching the disc space. The anterior C1 arch was divided bilaterally just lateral to the dens, followed by division of the alar ligaments, transverse ligament, apical ligament and tectorial membrane to release the dens from the clivus, thereby freeing the upper cervical spine from the CVJ.

**Figure 2:**
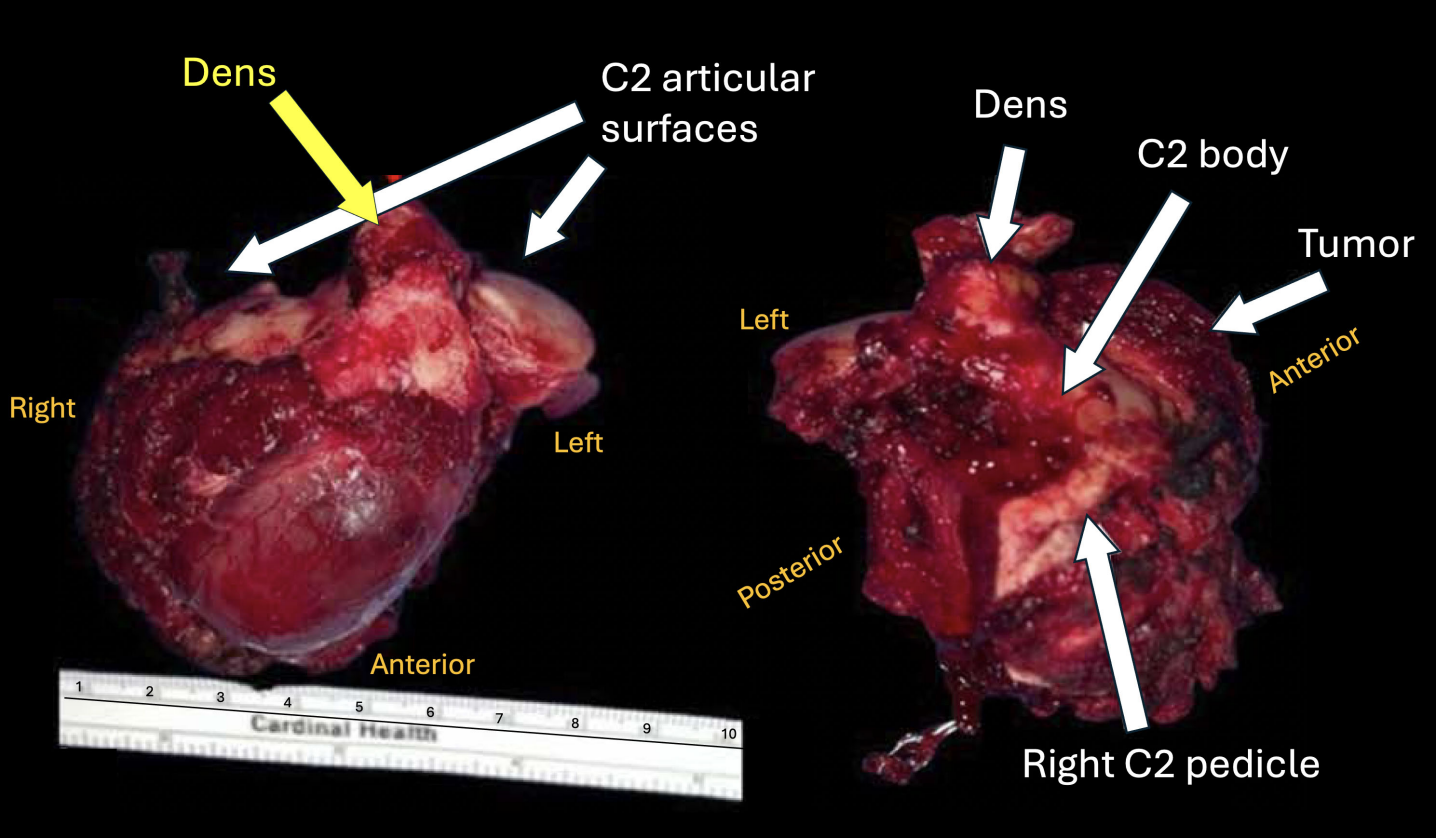
Images of en bloc tumor specimen incorporating the anterior arch of C1, C2 vertebral body and dens, right C2 pedicle, and C3 vertebral body.

The tumor was then mobilized en bloc – a mass that included the anterior arch of C1, the C2 vertebral body and dens, the right C2 pedicle, and the C3 vertebral body – and successfully delivered (**Figure 2**). Upon delivery of the tumor there was a loss of both extremity motor-evoked potentials and somatosensory-evoked potentials in all extremities. Mean arterial pressure was elevated to >100 mmHg and a bolus of dexamethasone was given. There was return of the right lower extremity for both monitoring modalities, but no improvement in the left lower extremity or bilateral upper extremities.

Anterior arthrodesis was performed using the patient-specific 3DPI. The 3DPI had been designed in advance of the surgical procedure based on anticipated operative defects using the proprietary PURI-TI methodology from the company DeGen Medical (Charleston, SC). The time from conception and planning of the 3DPI to its implantation during surgery was 14 days. This 3DPI spanned from the bilateral C1 lateral masses to the superior endplate of C4. The cage itself was one piece with a lateral portion designed to incorporate autologous bone graft. The 3DPI was superiorly secured to the C1 lateral masses via two screws (one 4x14 mm, the other 4x18 mm) and inferiorly secured to C4 via two screws (both 4x16 mm).

A vascularized myoosseous fibular autograft had been harvested by otolaryngology and was placed through the specially designed component of the 3DPI. This graft spanned from the right C1 lateral mass to the superior right C4 transverse process. The graft was secured by a plate with screws in the fibula and the right C4 vertebral body. The graft vasculature was anastomosed to right lingual artery and facial vein. The right mandible was resecured via plates. The neck was closed in layers. The patient was then carefully flipped prone for stage 3.

Stage 3 involved an occiput-to-C6 posterior instrumentation and fusion. Using freehand technique, the occipital plate/screws and lateral mass screws at C4, C5, and C6 were placed. Two 4 mm cobalt chrome rods were cut to span the construct. Remaining fibular autograft was split in half and used to span the posterior osseous defect from the occiput to the lateral masses of C6 bilaterally. Allograft was placed for additional augmentation of the arthrodesis. The posterior neck was then closed.

Upon weaning sedation in the operating room, the patient was able to move all extremities at least antigravity despite the loss of motor- and somatosensory-evoked potentials. He was transferred still intubated but in stable condition to the intensive care unit. In aggregate, the three consecutive stages required a total operative time of approximately 29 hours. Estimated blood loss was approximately 3, 000 mL. Surgical pathology demonstrated negative margins.

### Postoperative course

Postoperatively, he remained ventilator-dependent for a prolonged period. He received a tracheostomy on postoperative day 13 and ultimately weaned off the ventilator entirely on postoperative day 47. During this time, he developed a right pulmonary embolism for which he had an IVC filter placed and was eventually started on therapeutic anticoagulation. He had a gastrostomy tube placed approximately 1.5 months after surgery due to swallowing difficulties. Two months after surgery, he was walking up to 1000 feet with therapy and was discharged to a rehabilitation facility. At the time of discharge, his neurological exam was notable for right tongue deviation, 3/5 strength in the right deltoid, 4/5 strength in the right biceps/triceps, and 4+/5 strength in the left deltoid.

**Figure 3:**
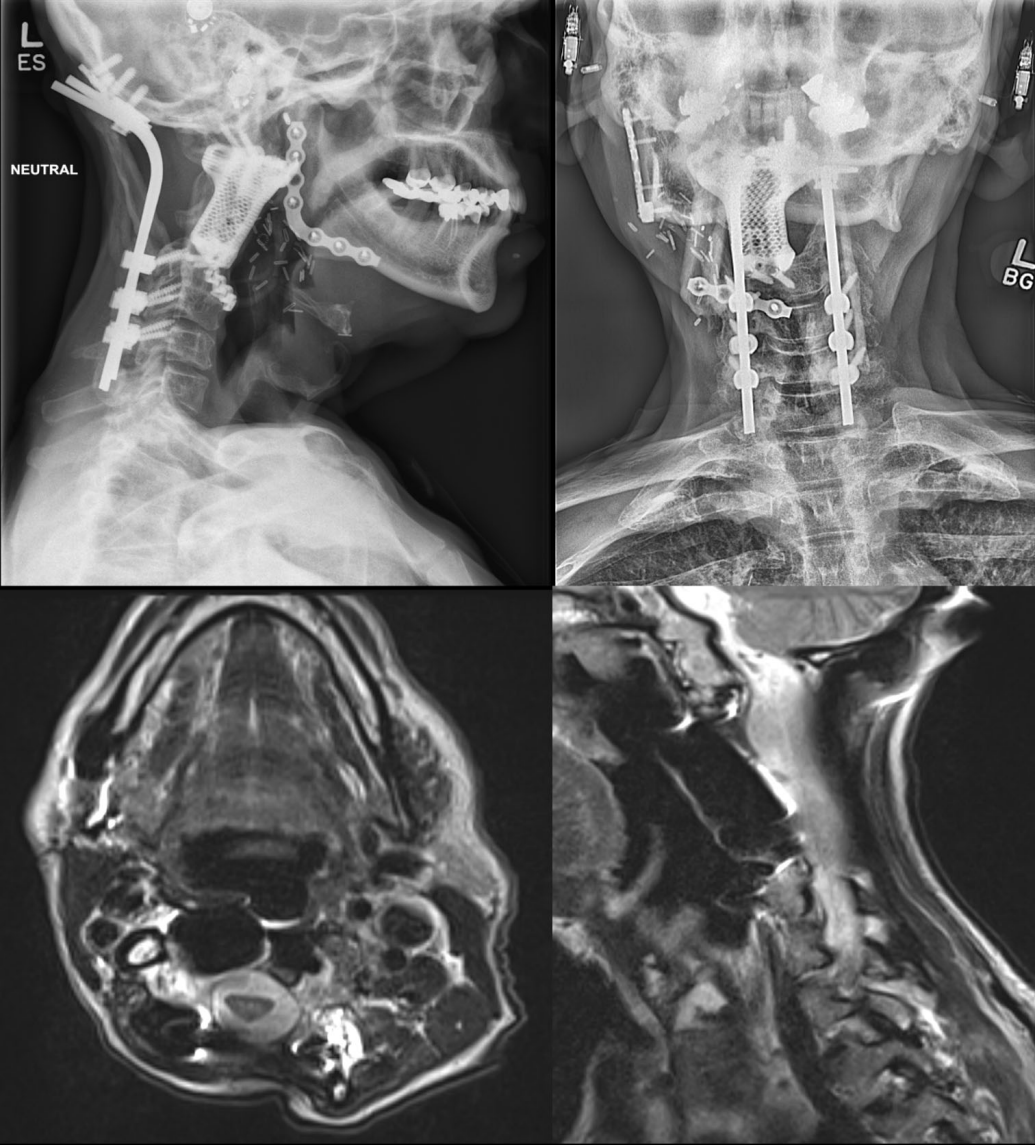
Postoperative imaging at two-year follow-up, including cervical radiographs that demonstrate maintained alignment and positioning of the 3DPI (upper images) and T2-weighted MRI that demonstrates the operative cavity without signs of recurrence (bottom images).

At his 6-month follow up, he no longer necessitated a tracheostomy and was full strength throughout all extremities. He was able to improve his swallowing ability and have his gastrostomy tube removed approximately 1.5 years after surgery. Most recent postoperative radiographs, taken at 2-year neurosurgical follow-up, showed the instrumentation and 3DPI in adequate position with unchanged alignment (**Figure 3**). He remains without evidence of recurrence, full strength in the extremities, and with good functional status over two years after surgery.

## Assessment of case through CLiMR framework

The authors first published on the Column, Line-of-Sight, Margins, Ring (CLiMR) framework in 2025 (10). CLiMR consists of four domains as described in **Table 1**. The Column domain describes the osteotomies and diskectomies required to release the tumor from the spinal column. The Line-of-Sight domain describes the surgical approach required to obtain circumferential access to the tumor. Points for different aspects of the Column and Line-of-Sight domains may be additive depending on the complexity of the surgery. The Margin domain describes the structures that had to be sacrificed to obtain negative margins. Unlike the Column or Line-of-Sight components, the most complex aspect of the Margin domain defines the points assigned to it. That is, if both vessels (4 points) and eloquent nerves (6 points) were sacrificed, then the Margin score would be 6 points (not 10 points). The Ring domain details the axial osseous cuts used to open the spinal canal for safe rotation and removal of the vertebral body. The size of the axial window created is proportional to the circumferential tumor involvement of the vertebral body. A smaller window carries inherently higher neurological risk as the neurological elements within the canal must be delivered through this small window. As with the Margins domain, points for this section are defined by the single most complex surgical maneuver. The minimum CLiMR score is 3.5 and, though it has a theoretical very high maximum score, nearly all cases will have scores <14.

**Table 1:** Explanation of CLiMR classification domains to evaluate en bloc tumor resections.

CLiMR domains:			
Column (C)	Line-of-Sight (L)	Margins (M)	Ring (R)
Sagittal split osteotomy (0.5 per vertebrae)	Posterior approach (1)	Noneloquent nerves (1)	Bilateral laminectomy or bilateral facetectomy (1)
Vertebrectomy/diskectomy (1 per vertebrae)	Each additional approach required (+1)	Muscle (2)	Hemilaminectomy or unilateral facetectomy (2)
Radical diskectomy (+0.5)	Special osteotomy or complex dissections (e.g. mandibulectomy) (+1)	Pleura/ribs/chest wall (3)	Bilateral pedicle base amputation (3)
		Vessels (4)	Unilateral pediculectomy (4)
		Organs/viscera (5)	
		Eloquent nerves (6)	
		Limb sacrifice (7)	
CLiMR scores by domain:			
Category	Components		Domain Score
Column (C)	C2 vertebrectomy: 1 point C3 vertebrectomy: 1 point C3 Sagittal split osteotomy: 0.5 point		2.5
Line-of-Sight (L)	Posterior approach: 1 point Anterior approach: +1 additional point Right mandibulectomy: +1 additional point		3
Margins (M)	Eloquent nerve resection (right C3 nerve root): 6 points		6
Ring (R)	Left C2 pediculectomy		4
			Total CLiMR Score: 15.5

The scores in parentheses represent the complexity or extent of surgical procedure required. The domain scores for this case sum to a total score of 15.5.

Applying the CLiMR framework to this upper chordoma case yielded a total score of 15.5 (**Table 1**), indicating that it was an exceptionally anatomically complex and technically demanding resection. Stratifying the procedure by CLiMR domain, it received 2.5 Column points (C2 and C3 vertebrectomies and C3 sagittal split osteotomy); 3 Line-of-Sight points (posterior and anterior approaches and right mandibulectomy); 6 Margin points (Right C3 nerve root sacrifice, considered eloquent given its contribution to diaphragmatic innervation); and 4 Ring points (left C2 pediculectomy). This unusually high CLiMR score highlights the complexity and risk associated with the anatomy of the CVJ and upper cervical spine. It also is consistent with the CLiMR authors’ findings that CLiMR score correlated with case length, blood loss, postoperative hospital length-of-stay, and postoperative complications (10). That said, we were able to obtain negative margins intraoperatively and the patient remains recurrence-free over two years postoperatively – very fortunate outcomes given the case difficulty.

## Discussion

Chordomas of the upper cervical spine are among the most challenging cases in spinal oncology and often require navigating complex CVJ anatomy. In this report, we describe a successful three-stage en bloc resection of a C2–3 chordoma and apply the novel CLiMR framework to characterize the anatomic and technical complexity of the procedure in a systematic and reproducible manner.

The oncologic rationale for en bloc resection of spinal chordomas is well-established (2–4, 11–13). These tumors are locally aggressive, resistant to conventional chemotherapy, and carry a significant risk of recurrence when treated with intralesional excision. High-dose radiotherapy reduces local recurrence rates when administered as an adjunct following en bloc resection, but its efficacy is dependent on negative surgical margins (14). The cervical spine en bloc literature remains sparser than for thoracolumbar or sacropelvic chordomas, and more studies are essential to improve outcomes and help surgeons navigate the neurovascular and biomechanical demands that make the upper cervical and CVJ regions uniquely hazardous.

Prior articles on high cervical chordoma en bloc resections are limited to a small number of case reports and short series, reflecting both its relatively low incidence and the need for extensive multidisciplinary infrastructure to undertake these procedures (5–8, 15–19). The present case is illustrative of the scope of preparation that such surgeries require. This included preoperative catheter angiography with balloon test occlusion of the vertebral arteries, a critical preliminary step in cases where vertebral artery sacrifice may be needed. Meticulous surgical planning was undertaken for weeks preceding the case, and a custom 3DPI was designed based on anticipated operative defects. Intraoperative loss of evoked potentials upon tumor delivery, followed by meaningful neurological recovery, underscores the physiological precariousness of this region and the importance of neuroprotective measures including permissive hypertension during the most critical moments of the procedure.

The removal of bony/soft tissue elements from C1–3, including the atlantoaxial articulation and occipitocervical ligamentous complex, created a large structural defect that had to be bridged to restore craniocervical alignment, preserve load-bearing mechanics, and promote arthrodesis. The integration of patient-specific additive manufacturing into surgical planning for complex oncologic reconstructions has emerged as a valuable tool in recent years, enabling implants that conform precisely to patient-specific bony anatomy and accommodate the unique geometry of multilevel defects that cannot be reliably spanned by off-the-shelf devices (20–25). In the present case, the 14-day turnaround from implant conception to intraoperative use highlights the logistical feasibility of this approach within a relatively time-sensitive setting, though it is possible to design implants on an even faster timescale (22). At two-year follow-up, the construct remained in satisfactory position with stable alignment, demonstrating the durability of the reconstruction strategy (**Figure 3**).

An important contribution of this report is the application of the CLiMR framework to a case at the far end of en bloc surgical complexity (**Table 1**). The CLiMR framework was developed by our group as a structured, domain-specific classification system incorporating four component scores to allow surgeons to relay the relative complexity and operative invasiveness of spinal oncology cases in a standardized and easily interpretable manner. Critically, while the Column and Line-of-Sight scores are additive across all components, the Margin and Ring scores are each defined by the single most complex maneuver performed in that domain, a deliberate design choice that prevents score inflation from redundant anatomic complexity and reflects the surgical reality that the most dangerous element of an operation, not the sum of routine steps, often determines its overall risk profile.

The authors found that higher CLiMR scores, particularly those >10, were associated with higher rates of positive margins and local tumor recurrence in addition to greater operative time, estimated blood loss, hospital length-of-stay, and postoperative complications (10). Describing this current case as having a CLiMR score of 15.5, then, contextualizes the risk profile of the operation within an evidence-based framework, rather than relying on narrative description alone. It facilitates multidisciplinary operative planning by requiring the surgical team to prospectively enumerate the specific maneuvers anticipated for each CLiMR domain (e.g. vascular/nerve root sacrifice, need for mandibulectomy). Moreover, CLiMR has potential value for inter-institutional benchmarking and quality improvement, enabling more meaningful comparisons of outcomes across en bloc resection case series that may otherwise appear superficially similar but differ in underlying anatomic complexity.

Of note, this case involves the paradox of an atypically high CLiMR score with optimal oncologic and functional outcomes for the patient. This suggests that while CLiMR score is a robust predictor of procedural complexity and risk, it should be understood as a measure of anatomic challenge rather than a deterministic predictor of outcomes. Expert center volume and multidisciplinary infrastructure almost certainly mitigate the risks inherent to high CLiMR score resections (26, 27). Future prospective CLiMR studies are necessary to better quantify how institutional experience and surgical technique modify the relationship between CLiMR complexity and postoperative outcomes.

This report carries the limitations of a single-institution case report. The generalizability of both the operative technique and the outcomes described is affected by our high-volume academic center with specialized oncologic neurosurgical expertise. The CLiMR framework has not yet been applied prospectively to a large series of cervical en bloc resections, and the scoring conventions for certain upper cervical anatomic maneuvers may require domain-specific refinement as additional cases are accumulated. Furthermore, the long-term durability of patient-specific titanium 3DPI implants of the CVJ remains an area of active investigation, and longer follow-up will be required to fully characterize fusion rates and implant longevity in this biomechanically demanding location.

## Conclusions

In an illustration of the anatomic and technical complexities inherent to the CVJ, we describe the en bloc resection of an upper cervical spine chordoma through a multilevel spondylectomy with reconstruction using a custom 3DPI. This procedure, carried out over three stages, has resulted in negative surgical margins and promising neurological and oncologic outcomes at two years. Application of the CLiMR framework to this case yields a score of 15.5, an exceptionally high score that highlights the complexity of upper cervical en bloc resections and demonstrates the utility of CLiMR as a standardized tool for operative characterization and risk communication, though further studies of the upper cervical spine and CVJ are required to further establish CLiMR’s benefits.

## Data Availability

The raw data supporting the conclusions of this article will be made available by the authors, without undue reservation.
